# Cosmopolitanism and Biogeography of the Genus *Manganonema* (Nematoda: Monhysterida) in the Deep Sea

**DOI:** 10.3390/ani1030291

**Published:** 2011-09-05

**Authors:** Daniela Zeppilli, Ann Vanreusel, Roberto Danovaro

**Affiliations:** 1 Department of Marine Sciences, Polytechnic University of Marche, Via Brecce Bianche, 60131, Ancona, Italy; E-Mail: d.zeppilli@univpm.it; 2 Marine Biology Research Group, Ghent University, Krijgslaan, 9000 Ghent, Belgium; E-Mail: ann.vanreusel@ugent.be

**Keywords:** deep sea, biogeography, cosmopolitanism, nematodes, *Manganonema*

## Abstract

**Simple Summary:**

The deep sea comprises more than 60% of the Earth surface, and likely represents the largest reservoir of as yet undiscovered biodiversity. Nematodes are the most abundant taxon on Earth and are particularly abundant and diverse in the deep sea. Nevertheless, knowledge of their biogeography especially in the deep sea is still at its infancy. This article explores the distribution of the genus *Manganonema* in the deep Atlantic Ocean and Mediterranean Sea providing new insights about this apparently rare deep-sea genus.

**Abstract:**

Spatial patterns of species diversity provide information about the mechanisms that regulate biodiversity and are important for setting conservation priorities. Present knowledge of the biogeography of meiofauna in the deep sea is scarce. This investigation focuses on the distribution of the deep-sea nematode genus *Manganonema*, which is typically extremely rare in deep-sea sediment samples. Forty-four specimens of eight different species of this genus were recorded from different Atlantic and Mediterranean regions. Four out of the eight species encountered are new to science. We report here that this genus is widespread both in the Atlantic and in the Mediterranean Sea. These new findings together with literature information indicate that *Manganonema* is a cosmopolitan genus, inhabiting a variety of deep-sea habitats and oceans. *Manganonema* shows the highest diversity at water depths >4,000 m. Our data, therefore, indicate that this is preferentially an abyssal genus that is able, at the same time, to colonize specific habitats at depths shallower than 1,000 m. The analysis of the distribution of the genus *Manganonema* indicates the presence of large differences in dispersal strategies among different species, ranging from locally endemic to cosmopolitan. Lacking meroplanktonic larvae and having limited dispersal ability due to their small size, it has been hypothesized that nematodes have limited dispersal potential. However, the investigated deep-sea nematodes were present across different oceans covering macro-scale distances. Among the possible explanations (hydrological conditions, geographical and geological pathways, long-term processes, specific historical events), their apparent preference of colonizing highly hydrodynamic systems, could suggest that these infaunal organisms are transported by means of deep-sea benthic storms and turbidity currents over long distances.

## 1. Introduction

Nematodes are the most abundant metazoans on Earth [1], they are ubiquitous in marine habitats [2] and their numerical dominance increases in the deep sea (up to >90%) with increasing water depth [3-6]. This phylum is characterized by a very high species number: more than 20,000 have been described [7], and, among them, about 4,000-5,000 are marine species. Meanwhile, the number of marine nematode species is evaluated from 10,000 to 20,000 species according to the most conservative estimation [8]. In particular, deep-sea nematode assemblages are characterized by a very high α-diversity and evenness, which is comparable with that of the tropical sub-littoral zone [9]. Even if the deep sea comprises about 91% of the ocean seafloor, investigations focused on the study of nematode diversity have been so far performed on a cumulative area of only 60–70 m^2^ [10]. Moreover, because of the small size, the taxonomy of deep-sea nematodes is difficult [11] and information on their distribution remains extremely scant [12].

Spatial patterns of species diversity provide information about the mechanisms that regulate biodiversity [13,14] and are important for setting conservation priorities [15]. Nevertheless, the present knowledge of the biogeography of deep-sea meiofauna is limited [16,17]. Cosmopolitan species are known among shallow-water nematodes [18]. Cosmopolitanism is known also for some deep-sea nematode genera, whilst only a few genera have been reported to inhabit a single habitat [19]. The cosmopolitanism of most genera does not necessarily apply to all species of the genus; and several studies suggest the presence of large differences in species distribution at both local and regional scale [10,12,19-22]. Studies on deep-sea nematode species distribution over large areas demonstrate that the distribution of some species is widespread [7,12,23]. For example, among the nine species of the genus *Dichromadora* described from the Weddell Sea, seven are found at more than one location and can cover distances in excess of 2,500 km [12].

This study focuses on the distribution of the deep-sea nematode genus *Manganonema* (Bussau 1993). This genus belong to the family Monhysteridae, recognized as one of the most under-investigated deep sea taxa [10]. *Manganonema* has been recorded in very low abundances (typically <2%), at water depths >600 m in many oceans [24-35]. As previously reported for the genera *Bathyeurystomina* and *Bathychaetosoma*, the genus *Manganonema* is apparently restricted to the deep sea. At present, the genus *Manganonema* contains six described species [29]. Different *Manganonema* species are typically reported from different sites, suggesting a potentially restricted distribution [29]. The aim of the present study is to investigate large-scale biogeography of the genus *Manganonema* in the deep sea. Forty-four specimens of eight different species were recorded from different Atlantic and Mediterranean regions. Four of the encountered species are new to science. The geographical and bathymetrical distributions of *Manganonema* genus and species were analyzed in order to test the cosmopolitan character of this genus also at species level.

## 2. Experimental Section

Specimens were collected from 20 distinct deep-sea sites from the Atlantic Ocean and the Mediterranean Sea at water depth ranging from 567 to 4,987 (Table 1). Sediment samples were collected from several oceanographic cruises in the North Atlantic at Rockall Through (R/V Pelagia, 2006), and off the Portugal coast (R/V Pelagia, 2006), in the Gulf of Guinea (R/V Pourquoi pas?, 2008), in the Eastern Atlantic and in the Western and in the Central Mediterranean Sea (R/V Urania, 2007, 2008 and 2009) and in the Eastern Mediterranean Sea (R/Vs Aegaeo and Universitatis, 2006 and 2007 respectively). Sediment samples were collected by multiple-, box- and interface-corers and preserved in buffered 4% formalin solution and stained with Rose Bengal.

Sediment samples were pre-screened through a 1,000-μm mesh net, and the organisms retained on a 20-μm mesh net. The fraction remaining on the latter sieve was re-suspended and centrifuged three times with Ludox HS40 (density 1.31 g cm^3^) and nematodes were mounted on slides after formalin-ethanol-glycerol treatment following the detailed protocols reported by Danovaro [36].

Drawings and photos were made on Leica DMLS microscope. Cobb formula, showing the distance of each character from anterior end was calculated for each specimen. Ratio a represents the body length divided by the body maximum width, ratio b represents the body length divided by the pharynx length, and ratio c represents body length divided by the tail length.

## 3. Results and Discussion

### 3.1. Distribution of the Genus Manganonema

**Figure 1:**
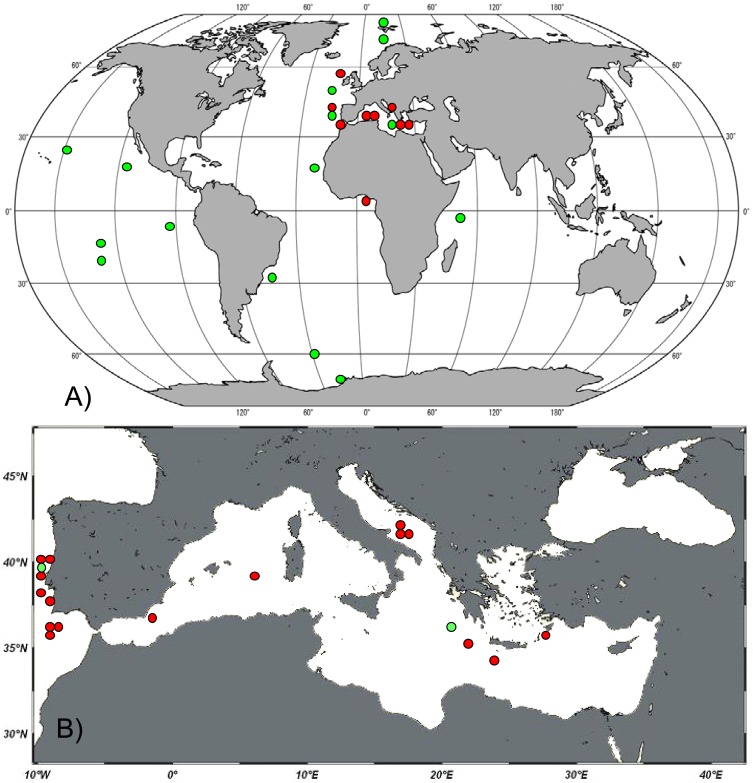
Geographic distribution of the genus *Manganonema* at global scale (**A**) and in the Mediterranean Sea (**B**). Red circles indicate locations of *Manganonema* records in the present study, green circles indicate *Manganonema* records available in literature (Antarctic Ocean [28,29], Arctic Ocean [33], Atlantic Ocean [29,30,32], Barents Sea [34], Pacific Ocean [24,26,27,29,35], Mediterranean Sea [31], Weddell Sea [25]).

We encountered the genus *Manganonema* in 20 different deep-sea sites from the Atlantic Ocean and the Mediterranean Sea (Table 1). The presence of the genus *Manganonema* was recorded in the Atlantic Ocean (Rockall Through, Portuguese Margin, Gulf of Cadiz and Gulf of Guinea) and in the Mediterranean Sea (Western, Central and Eastern). Figure 1(A) shows the distribution of *Manganonema* at the global scale, including the records available in literature. The new findings of *Manganonema ssp.* specimens together with literature data confirm the cosmopolitan character of this genus, which can be found in all oceans (Atlantic, Indian, Pacific, Arctic, and Antarctic) and at latitudes spanning from 70°N to 60°S. We discovered *Manganonema* in 16 specimens from the Mediterranean Sea (Figure 1(B)). Only one study previously documented the presence of this genus in the Mediterranean Sea [31]. *Manganonema* was recorded in three sites of the Eastern Mediterranean Sea, in three sites of the Southern Adriatic Sea and in two sites in the Western Mediterranean Sea. This demonstrates that the genus *Manganonema* is widespread in the deep-sea sediments of the Mediterranean Sea, from the Eastern part of the basin to the Atlantic Ocean. In addition half of the *Manganonema* species identified in the present study were reported in the Mediterranean Sea, including one species new to science. This supports the hypothesis that the deep Mediterranean basin provides a variety of environmental conditions that might favor the colonization of several rare species [22].

### 3.2. Bathymetric Distribution of the Genus Manganonema

**Figure 2:**
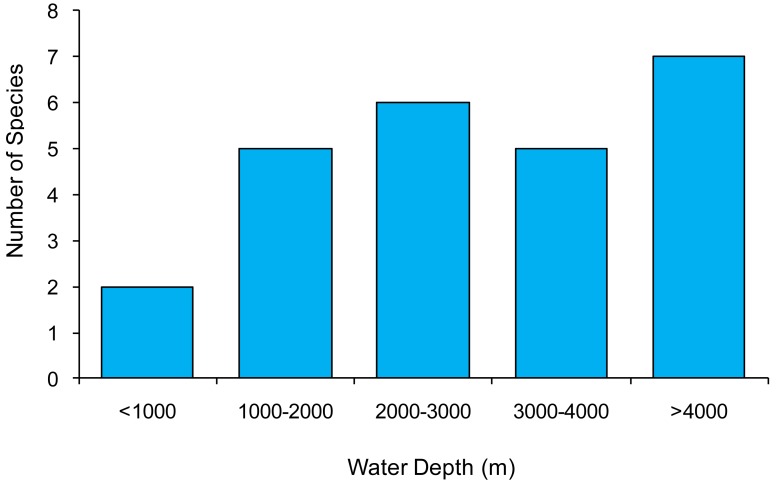
Bathymetric species richness of the genus *Manganonema*.

Our data show that *Manganonema* is present at depths ranging from 567 to 4,997 m. The highest diversity (7 species) was found at a water depth >4,000 m (Figure 2). *Manganonema* is considered a deep-sea genus as for *Bathyeurystomina* and *Bathychaetosoma* [29]. However, two species encountered at water depths <1,000 m in the North-east Atlantic Ocean (Rockall Trough, 567 m) and in the Central Mediterranean Sea (Southern Adriatic Sea, between 590–824 m) indicate that this genus can be piezotolerant. The only other study that described the presence of *Manganonema* at shallow depths (600 m) was in the Weddell Sea [29], this was explained by the fact that the peculiar conditions of this marine system could be compared with those of the deepest ocean, and thus offer the opportunity to this genus to colonize shallow sediments. Accordingly, here we discovered *Manganonema* in shallow sites that are characterized by habitats such as cold-water corals, seamounts, erosional features and different physical conditions (e.g., bottom temperature of the Mediterranean Sea and Atlantic Ocean), which share the presence of high hydrodynamics (*i.e.*, strong bottom currents).

### 3.3. Habitats Inhabited by Manganonema

The genus *Manganonema* was previously reported from diverse deep-sea habitats, from the Arctic to the Antarctic Ocean, in reduced ecosystems (hydrothermal vents and mud volcanoes), in submarine canyons and in polymetallic nodule deposits [24,26-29,32-35]. Here we discovered *Manganonema* in cold-water coral habitats and in seamounts. Furthermore, we recorded *Manganonema* also in different morphologies and sedimentary features related to submarine landslides, unstable slopes or seafloor erosion (e.g., mud waves and furrows). This study reinforces the idea that the genus *Manganonema* is able to colonize a very different variety of deep-sea habitats.

### 3.4. Distribution of the Manganonema Species

Some meiofaunal species (including nematodes) display a wide spatial distribution [37] and a trans-oceanic dispersal of free-living marine nematodes is not rare [18]. Most dominant deep-sea nematode genera are considered to be cosmopolitan, but at the species level little is known about the geographic distribution of their species.

**Figure 3:**
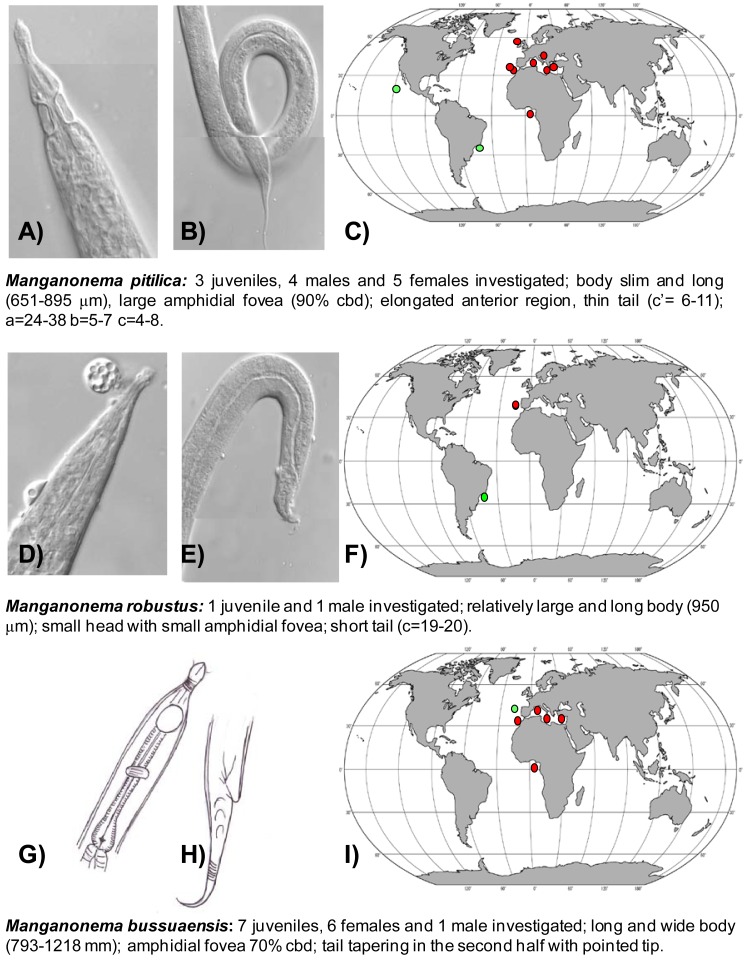
Pictorial key for *Manganonema pitilica* (**A**–**C***), Manganonema robustus* (**D**–**F**) and *Manganonema bussuaensis* (**G**–**I**) with the representation of the anterior and posterior ends, geographical distribution (red circles indicate records in the present study, green circles indicate records available in literature) and short diagnosis of each species. Reported are ratio a = body length divided by maximum body diameter, b = body length divided by pharyngeal length, c = body length divided by tail length, c′ = tail length divided by anal body diameter, cbd = corresponding body diameter.

**Figure 4:**
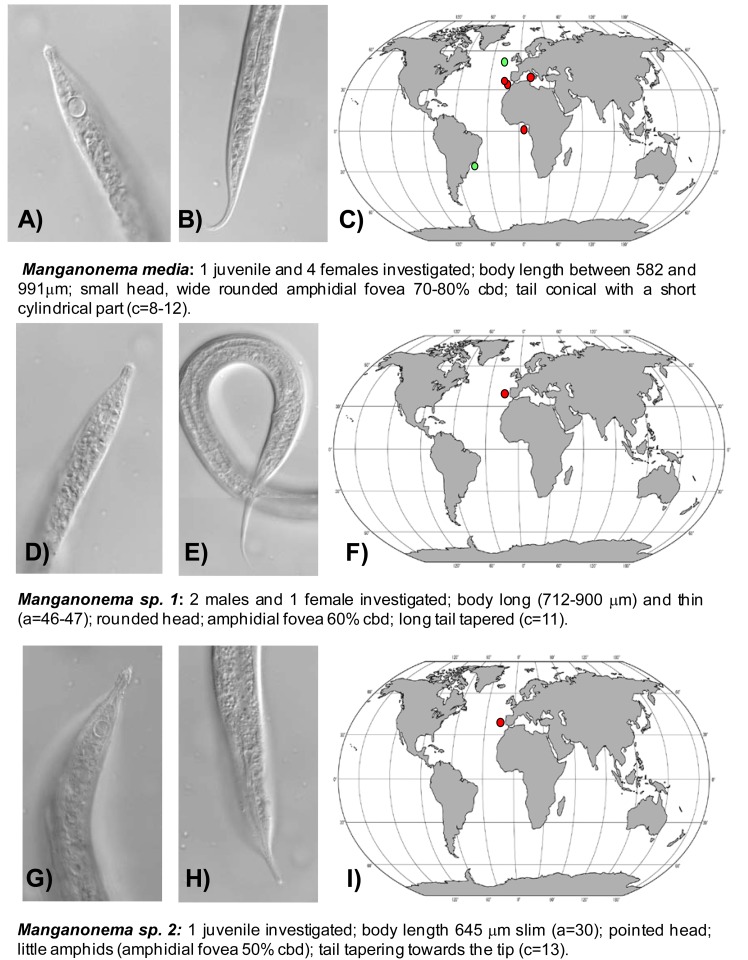
Pictorial key for *Manganonema media* (**A**–**C**), *Manganonema sp. 1* (**D**–**F**) and *Manganonema sp. 2* (*putative species*) (**G**–**I**) with the representation of the anterior and posterior ends, geographical distribution (red circles indicate records in the present study, green circles indicate records available in literature) and short diagnosis of each species. Reported are ratio a = body length divided by maximum body diameter, b = body length divided by pharyngeal length, c = body length divided by tail length, c′ = tail length divided by anal body diameter, cbd = corresponding body diameter.

**Figure 5:**
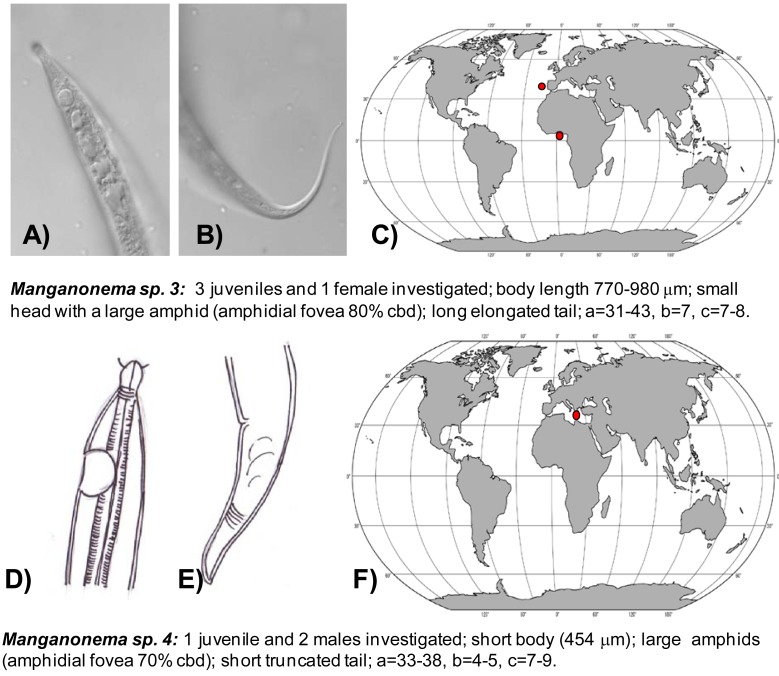
Pictorial key for *Manganonema sp. 3* (*putative species*) (**A**–**C**) and *Manganonema sp. 4* (**D**–**F**) with the representation of the anterior and posterior ends, geographical distribution (red circles indicate records in the present study, green circles indicate records available in literature) and short diagnosis of each species. Reported are ratio a = body length divided by maximum body diameter, b = body length divided by pharyngeal length, c = body length divided by tail length, c′ = tail length divided by anal body diameter, cbd = corresponding body diameter.

The first exhaustive analysis of the *Manganonema* species hypothesised a restricted distribution of this genus [29]. However, subsequent studies revealed that some species of *Manganonema* are not endemic [35]. In the present study 44 specimens of *Manganonema* belonging of eight species were analysed. Although a description of the new *Manganonema* species is beyond the scope of this work, a pictorial key is provided in Figure 3, Figure 4 and Figure 5. Species represented only by females and juveniles were considered as putative species (*Manganonema sp.2* and *Manganonema sp.3*).

*Manganonema pitilica* (Fonseca, Decraemer, Vanreusel, 2006) was previously found in the Pacific Ocean and in the South-west Atlantic Ocean (Figure 3(A,B,C)) [29,35]. We found this species in the North-east Atlantic (Rockall Trough, Portuguese Margin, Gulf of Cadiz, and in Gulf of Guinea) and in the Western, Central and Eastern Mediterranean Sea at all investigated depths. *M. pitilica* is widely distributed and able to colonize different habitats from manganese nodule areas [35] to seamounts and cold-water coral ecosystems.

*Manganonema robustus* (Fonseca, Decraemer, Vanreusel, 2006) was discovered in the North-east Atlantic Ocean (Portuguese Margin) at water depths between 2,000 and 5,000 m. This species was previously reported only in the Southwest Atlantic, off the Brazilian coasts (Figure 3(D,E,F)) [29].

*Manganonema bussuaensis* (Fonseca, Decraemer, Vanreusel, 2006) was found in the North-east Atlantic Ocean (Gulf of Cadiz and Gulf of Guinea) and in Western, Central and Eastern Mediterranean Sea from 714 to 4,400 m water depth. This species was previously reported only in the North Atlantic at 2,000 m water depth (Figure 3(G,H,I)) [29].

*Manganonema media* (Fonseca, Decraemer, Vanreusel, 2006) was encountered in the North-east Atlantic Ocean (Portuguese Margin, Gulf of Cadiz and Gulf of Guinea) and in Western Mediterranean Sea at water depth between 2000 and 5000 m. This species was previously reported only in the South-west Atlantic, off the Brazilian coasts and in the North-east Atlantic, at Goban Spur (Figure 4(A,B,C)) [29].

*Manganonema sp. 1* was found in the North-east Atlantic Ocean (Portuguese Margin) at 2,000 and 4,000 m water depth (Figure 4(D,E,F)).

*Manganonema sp. 2* (putative species) was encountered in the North-east Atlantic Ocean (Portuguese Margin) at 3475 m water depth (Figure 4(G,H,I)).

*Manganonema sp. 3* (putative species) was reported in the North-east Atlantic Ocean (Portuguese Margin and Gulf of Guinea) at water depth between 1,700 and 5,000 m (Figure 5(A,B,C)).

*Manganonema sp. 4* was encountered in the Eastern Mediterranean Sea at 3000 m water depth (Figure 5(D,E,F)).

Finally, in order to provide a complete review on biogeography and distribution of the *Manganonema* genus, the distribution of the remaining described species (not encountered in this study) are presented.

*Manganonema antarctica* was reported in two different sites of the Southern Ocean: the Weddell Sea and the South Sandwich Trench at water depth between 600 and 4,000 m [29].

*Manganonema microchepalum* was reported in the Peru Basin, Central Pacific Ocean at 4,000 m water depth [26,29].

The results of this study suggest that *Manganonema* species are not isolated as previously supposed [29]. Based on distribution patterns of single species we observed that *Manganonema* species can be either apparently restricted or widespread and one *Manganonema* species (*M. pitilica*) is a possible cosmopolitan species. Furthermore, at some sites, up to 3 different species were found to coexist, supporting the hypothesis of a trophic specialization and resulting in an important contribution to the local species richness [29].

### 3.5. Dispersal Mechanisms of Deep-Sea Nematodes

Some *Manganonema* species are apparently confined to specific areas, while others have a much wider spatial distribution. These contrasting dispersal strategies may be linked to their behaviour or life cycle. Adult nematodes are expected to have limited dispersal capabilities and hence a reduced gene flow, making restricted species distributions more plausible [38]. However some deep-sea nematodes have been demonstrated to have important dispersal capabilities [7]. The continental and cosmopolitan distributions of many meiofaunal taxa appear to result from a variety of dispersive mechanisms. Several geological, geographical and hydrodynamic pathways (*i.e.*, passive erosive suspension, active emergence, seamounts, sediment transports) and natural/anthropogenic rafting should be considered [2]. Adult benthic organisms can be transported also within moving sediment, for example benthic storms, submarine landslides and turbidity currents can carry large amounts of sediment over long distances [39,40], with an additional effect of defaunating large areas that are then free for re-colonisation. In addition to the adult macrofaunal organisms, larvae and buoyant eggs can stay in the water column for a long time, extending their potential dispersal [41]. The lack of meroplanktonic larvae or buoyant eggs does not allow nematodes to use these strategies for their dispersal, but sediment resuspension due to bottom currents or benthic storms can certainly contribute to the dispersal of the nematodes. This could apply particularly to the genus *Manganonema* that apparently prefers habitats characterized by high hydrodynamic conditions.

## 4. Conclusions

*Manganonema* is a cosmopolitan genus, inhabiting all oceans (Atlantic, Indian, Pacific, Arctic, and Antarctic) and spanning across a large variety of deep-sea habitats. This genus can be found at latitudes ranging from 70°N to 60°S. We report here that the genus *Manganonema* is widespread also in both basins of the Mediterranean Sea. Fifty percent of the species encountered were present also in the Mediterranean Sea, and one of these is new to science. *Manganonema* is a deep-sea genus characterized by the highest diversity at depths >4,000 m. However, this genus is able to colonize also sediments at a water depth <1,000 m, where peculiar environmental conditions exist, for example the presence of strong near bottom currents.

Finally, the results of this study show that *Manganonema* showed remarkable differences among species, spanning the entire range between endemic to cosmopolitan. This genus shows the co-presence of several species, indicating a potential local diversity. Moreover, half of the species encountered are new to science, revealing the potential and hidden biodiversity of deep-sea nematodes. In spite of the evidence that adult nematodes have limited dispersal capabilities, the deep-sea nematodes revealed a high dispersion. A possible explanation is that infaunal organisms, and thus also nematodes, can be transported along with surface sediments by turbidity currents and benthic storms over long distances.

## Figures and Tables

**Table 1:** List of the sampling sites. Reported are latitude longitude, water depth, region, habitat and number of investigated *Manganonema* specimens for each station.

**Station**	**Latitude**	**Longitude**	**Water depth (m)**	**Region**	**Habitat**	***Manganonema* specimens**
1	55°29.71′N	15°48.56′W	567	North-east Atlantic Ocean (Rockall Through)	Cold-water corals	1
2	41°52.54′N	17°00.47′E	590	Central Mediterranean Sea (Southern Adriatic Sea)	Erosional structure (mud wave)	2
3	41°47.34′N	17°01.85′E	714	Central Mediterranean Sea (Southern Adriatic Sea)	Erosional structure (furrow)	1
4	41°31.88′N	17°25.15′E	824	Central Mediterranean Sea (Southern Adriatic Sea)	Dauno seamount	1
5	02°57.26′N	06°49.51′E	1,671	North-east Atlantic Ocean (Gulf of Guinea)	Open continental slope	3
6	02°56.87′N	06°49.44′E	1,701	North-east Atlantic Ocean (Gulf of Guinea)	Submarine landslides	3
7	34°40.26′N	24°07.66′E	1,998	Eastern Mediterranean Sea	Unstable continental slope	1
8	37°50.00′N	09°45.00′W	2,130	North-east Atlantic Ocean (Portuguese Margin)	Open continental slope	2
9	36°47.38′N	00°29.06′W	2,689	Western Mediterranean Sea	Open continental slope	2
10	35°00.34′N	08°16.27′W	2,788	North-east Atlantic Ocean (Gulf of Cadiz)	Open continental slope	6
11	39°18.80′N	06°04.25′E	2,855	Western Mediterranean Sea	Open continental slope	1
12	35°08.33′N	20°50.88′E	3,000	Eastern Mediterranean Sea	Open continental slope	6
13	35°57.64′N	28°17.63′E	3,009	Eastern Mediterranean Sea	Open continental slope	2
14	40°10.00′N	09°59.99′W	3,475	North-east Atlantic Ocean (Portuguese Margin)	Open continental slope	1
15	39°13.74′N	10°59.00′W	4,060	North-east Atlantic Ocean (Portuguese Margin)	Unstable continental slope	3
16	34°37.49′N	09°17,00′W	4,335	North-east Atlantic Ocean (Gulf of Cadiz)	Open continental slope	1
17	34°32.75′N	09°45.38′W	4,381	North-east Atlantic Ocean (Gulf of Cadiz)	Open continental slope	1
18	34°08.02′N	09°33.28′W	4,385	North-east Atlantic Ocean (Gulf of Cadiz)	Open continental slope	3
19	40°10.01′N	10°59.99′W	4,902	North-east Atlantic Ocean (Portuguese Margin)	Open continental slope	2
20	37°50.01′N	11°00.01′W	4,987	North-east Atlantic Ocean (Portuguese Margin)	Open Continental Slope	2
